# Essential Healthcare Services in the Face of COVID-19 Prevention: Experiences from a Referral Hospital in Ethiopia

**DOI:** 10.4269/ajtmh.20-0464

**Published:** 2020-08-05

**Authors:** Seid Getahun Abdela, Abel Balcha Berhanu, Leul Mesfin Ferede, Johan van Griensven

**Affiliations:** 1 Department of Internal Medicine, College of Medicine and Health Sciences, Wollo University, Dessie, Ethiopia;; 2 Department of Surgery, CMHS, Wollo University, Dessie, Ethiopia;; 3 Department of Surgery, Dessie Referral Hospital, Dessie, Ethiopia;; 4 Institute of Tropical Medicine, Antwerp, Belgium

## Abstract

Globally, healthcare systems are facing the enormous challenge of the COVID-19 pandemic. Ethiopia is currently implementing different preventive measures to interrupt the transmission of SARS-CoV-2. The early effect of these preventive measures on essential healthcare service delivery is unknown. In this study, we looked at the number of essential healthcare visits over 8 weeks, 4 weeks before and 4 weeks after the implementation of preventive measures. During the implementation of these measures, patient flow decreased in all elements of essential healthcare service. The decline was dramatic for family planning (98%), emergency surgery (77%), and follow-up of chronic surgical conditions (70%). An understanding of the reasons behind the decrease in patient flow is urgently needed to design ways of sustaining essential care.

In January 2020, a novel virus, the SARS-CoV-2, was identified as the causative agent for a cluster of pneumonia cases initially detected in Wuhan City, Hubei Province, China.^1^ SARS-CoV-2, which causes the disease now named COVID-19, has subsequently spread throughout the globe. By May 12, 2020, the virus infected more than 4.2 million people and claimed more than 280,000 lives.^2^

Health systems in the world are facing a rapidly increasing demand generated by the COVID-19 pandemic. Africa is still in the early stage of the COVID-19 outbreak. The Ebola outbreak is a good example of how outbreaks may disrupt programs such as tuberculosis, HIV, and maternal and child health care, increasing mortality from preventable and treatable conditions.^3–8^ To mitigate the impact of COVID-19 on health systems, the WHO prepared a guideline on how to continue essential services during the COVID-19 pandemic. The guideline recommends continuation of essential services like vaccination, chronic disease follow-up, and maternal and child health care, taking into consideration the local context and extent of the outbreak. In areas with a relatively limited number of COVID-19 cases, the health system may have the capacity to maintain routine service delivery in addition to managing COVID-19 cases.^9^

Ethiopia reported the first COVID-19 case on March 13, 2020. As of May 11, 2020, 250 cases were reported. Like other countries, Ethiopia started to practice different prevention strategies after confirming the first case. These include partial and selective total lockdown, stopping mass praying, avoiding mass gatherings, and closing schools. Different task forces were established at the national and local levels. The Federal Ministry of Health planned to continue essential services during COVID-19 prevention and management. The Ethiopian essential health service package includes reproductive, maternal, neonatal, child, and adolescent health services; major communicable diseases; noncommunicable diseases; surgical care; and emergency and critical care.^10^

The Amhara regional state zones passed different preventive decisions, like stopping public transport and different physical distancing rules. Hospitals across the region stopped elective surgeries and stopped receiving nonemergency and self-referred patients. The hospitals restricted the number of patient attendants and spaced appointments of chronic illnesses. Dessie Referral Hospital, one of the facilities in the region serving more than 8 million people of the catchment area (East Amhara, part of Tigray, and Afar region), applied these measures starting from March 23, 2020. The transport restriction was lifted on April 16, 2020.

As of May 12, there was no confirmed case of COVID-19 in the hospital, and a single case was reported from a neighboring Afar region on April 23, 2020. The prevention of SARS-CoV-2 community transmission has been attributed to early and rigorous preventive measures. However, its effect on essential health care remains uninvestigated and not well known. Therefore, this study assessed the effect of prevention measures on essential healthcare services at Dessie Referral Hospital. A better understanding of the effect of preventive measures on the healthcare system will help to design ways of sustaining essential health services during the COVID-19 pandemic.

The study was performed in Dessie Referral Hospital. The hospital is located in Dessie town, South Wollo zone of northeast Ethiopia, 401 km from Addis Ababa. It has different departments such as emergency, ophthalmology, surgery, obstetrics and gynecology, child health, medical care, neonatal care, laboratory, psychiatry, pharmacy, and HIV care, and other clinics. The hospital is staffed with more than 800 healthcare and administrative workers. Annually, more than 300,000 patients are seen in the hospital.

Ten days after the first case notification on March 23, 2020, Dessie Referral Hospital applied different preventive measures. These measures include reducing the number of patient attendants to one, exempting vulnerable hospital staff from activities, and stopping service for nonemergency and self-referred patients. In addition, public transport was stopped until April 16, 2020. Isolation and treatment centers were established. Training on COVID-19 prevention and treatment was given for more than 100 hospital staff.

We collected data on the number of patients attending different essential healthcare services from the registers in the different services. These include maternal and child health (delivery, gynecology emergencies, neonatal, and pediatric emergencies), medical and surgical chronic illness follow-up, oncology, HIV/AIDS clinic, tuberculosis diagnosis, and medical and surgical emergencies. For each service, the weekly aggregate data were collected from February 24, 2020 to April 19, 2020, and the number of visits was compared before and after the implementation of COVID-19 preventive measures.

In all outpatient departments, the number of cases decreased during the implementation of preventive measures. The number of surgical and medical emergency visits decreased by more than 50%. The number of mothers delivering at the hospital remained relatively stable. However, the family-planning visits decreased by more than 95%. The number of pregnant women coming for antenatal care decreased by more than 50%. Likewise, the neonatal admission and other childhood emergency visits decreased by more than 70%. The units of blood at the hospital blood bank remained relatively stable. However, blood was not used for elective surgeries, and the number of emergency cases decreased. The number of samples sent for diagnosis of tuberculosis by GeneXpert decreased by more than 70% compared with the previous month peak (Table 1).

**Table 1:** Number of visits to essential healthcare–delivering units

Essential healthcare type	Number of visits before preventive measures (from February 24 to March 22)				Number of visits after preventive measures (from March 23 to April 19)			
	Week 1	Week 2	Week 3	Week 4	Week 5	Week 6	Week 7	Week 8
Outpatient								
Surgical	413	329	416	428	172	118	67	124
Medical	306	259	369	431	315	300	206	234
Psychiatry	201	193	241	181	184	137	101	157
Oncology	30	24	33	45	24	24	19	21
Antiretroviral therapy	622	486	565	678	625	610	399	505
Adult emergency	194	214	154	222	141	139	88	102
Emergency surgery	45	40	44	38	8	8	17	21
Maternal health								
Antenatal care	87	68	73	117	37	45	76	85
Family planning	77	68	73	8	1	3	1	1
Delivery	155	206	151	193	162	171	137	185
Gynecology emergency	35	46	30	26	28	18	26	20
Child health								
Neonatal intensive care unit	57	63	44	104	49	58	30	43
Pediatric emergency	132	157	159	144	88	36	36	90
Other								
Blood units in the hospital	88	94	83	86	99	97	70	88
Samples subjected for GeneXpert	57	45	43	60	43	12	14	22

In all components of the essential health services, the number of cases declined. In addition to the preventive measures, the public fear toward the disease may have contributed to the decreased flow. This may directly or indirectly increase mortality and morbidities from treatable and preventable conditions.

The number of patients coming for medical and surgical chronic illness follow-up decreased, and the decline was more pronounced in the surgical outpatient department. This may leave patients without timely evaluation and treatment, predisposing them for different medical and surgical complications. For example, malignancies may further grow and spread; patients with toxic goiter may develop different cardiac and central nervous system complications. In addition, chronic medical illness patients will run out of medications, epilepsy patients may experience seizures, diabetic patients may develop diabetic ketoacidosis, and cardiac and hypertensive patients may develop ischemic heart disease and cerebrovascular accidents. As to HIV/AIDS patients, appointments were spaced for all patients including new and those with treatment failure, which makes monitoring of adherence and side effects difficult.

The number of visits at the emergency service decreased substantially, particularly for urgent surgery. This may lead to delayed presentation and severe complications.

Almost all components of maternal and child health services suffered from a low case flow. The exception was the delivery service, which was relatively stable. This may be because of the strong programs and advocacies against home delivery. The family-planning service was nearly closed, serving less than five patients per week. This may increase the risk of unplanned pregnancies and related complications. The gynecology emergency care includes care for abortion. Preventive measures may have resulted in more patients seeking help from traditional healers, potentially leading to acute and chronic complications of abortion. Both neonatal and other childhood emergency visits have also decreased. Because Dessie Referral Hospital is the only referral hospital in the eastern Amhara region to deliver this service, there is a serious risk that the health of neonates and children needing this service is compromised. The number of tests for the diagnosis of tuberculosis also decreased substantially, potentially leading to delayed diagnosis.

This study has a number of important limitations. First, it was conducted in a single hospital. However, our findings are likely to reflect the status in the entire region, as all hospitals were applying similar preventive actions. Second, we were not able to collect information on the reasons behind the decrease in patient flow, and whether patients attended other healthcare facilities. This needs further studies with a larger number of health facilities.

The case flow of almost all essential healthcare services declined during COVID-19 preventive measures. Preventive procedures should be implemented considering the continuity of essential healthcare delivery. Public restrictions should be selective; emergency cases should still find a way to access hospitals. Early decentralization of chronic illness care to the primary healthcare level should be envisioned. Moreover, special attention should also be given to blood donation, HIV/AIDS care, and diagnosis of tuberculosis.
